# Stigma surrounding contraceptive use and abortion among secondary school teachers: A cross-sectional study in Western Kenya

**DOI:** 10.1016/j.conx.2021.100062

**Published:** 2021-02-07

**Authors:** Marielle E. Meurice, Beatrice Otieno, Jenny J. Chang, Marlene Makenzius

**Affiliations:** a University of California, Irvine, Department of Obstetrics and Gynecology, 333 City Blvd West, Suite 1400, Orange, CA, 92868, USA; b Kisumu Medical & Education Trust KMET, Kakamega Road P.O. Box 6805-40103, Kisumu, Kenya; c University of California, Irvine, Department of Medicine, School of Medicine, 301 Medical Surge II,Irvine CA 92697, USA; 4 A. Karolinksa Institutet, Department of Public Health Sciences, Global Public Health, Tomtebodavägen 18A, 171 77, Stockholm, Sweden

**Keywords:** Stigma, Abortion, Contraception, Comprehensive sexual education, Gender equality, Gender equity

## Abstract

**Objectives:**

Comprehensive sexual education plays an essential role in adolescent sexual and reproductive health and rights (SRHR). The study aim was to investigate Kenyan secondary school teachers’ attitudes toward girls associated with contraceptive use and abortion.

**Study design:**

We conducted a cross-sectional study in January 2018 among school teachers (*n =* 144) at 4 religiously affiliated suburban secondary schools in Kenya and used 2 validated Likert Scales (1–5) to capture contraception and abortion stigma.

**Results:**

Overall, 122 teachers responded (85%) (females, 57%; males 43%; age, 21–70 years [mean, 36]). Respondents associated contraceptive use with a promiscuous lifestyle (43%) that will encourage peers to do the same (51%). Respondents indicated that married women were more deserving of contraception than unmarried women (57%), a girl could not herself decide to use contraceptives (50%), and contraceptive use could impair future fertility (57%). Abortion was considered a sin (74%), shameful for the family (48%), a habit (34%), and a behavior that might encourage peers to do the same (51%). Many believed an abortion will lead to worse health (73%). Male and female teachers gave similarly distributed responses. Younger teachers were more likely to find abortion shameful (<29, 64%; 30–39, 39%; ≥40, 39%; *p =* 0.046). Contraception stigma and abortion stigma were highly correlated (r = 0.355, *p <* 0.001).

**Conclusions:**

Stigmatizing attitudes associated with contraceptive use and/or abortion were common among teachers in Western Kenya.

**Implications:**

Stigma may hinder the sexual and reproductive health and rights of students. Contraceptive use and abortion stigma need to be addressed in teacher education to ultimately improve health outcomes among adolescents.

## 1 Introduction

Approximately 464,690 abortions were performed in Kenya in 2012. Although abortion is legal, it is limited to saving a woman's life or health. This law has been in place since 2010, yet unsafe abortion persists at an unacceptably high level despite the change in legal status; 119,912 women sought care for complications related to induced abortion, 37% of these were severe complications. In 2012, the death rate was 266 of 100,000 unsafe procedures [1,2], and the abortion rate was approximately 48 of 1000 women of reproductive age, and 38 of 1000 women aged 15 to 19 years [3].

Among sexually active, unmarried adolescents, about half are using modern contraceptive methods and 1 in 5 have an unmet need for contraception [4]. In 2014, 15% of 15- to 19-year olds reported a live birth and by 19 years of age, 40% reported a live birth [4]. Given the high rates of teen pregnancies and unmet contraception needs, addressing adolescent sexual and reproductive health and rights has become a priority in Kenya.

In 2013, the Kenyan government announced an age-appropriate plan to scale up comprehensive sexuality education (CSE) in schools [5]. In Kenya, 86% of children attend primary school and 33% secondary school [4]. As most children will attend school, it is the place where sexual education might have a significant impact on sexual and reproductive health and rights (SRHR) [6]. Despite governmental efforts to bolster CSE, a study from 2015 revealed the lessons focused on abstinence, and portrayed sex and abortion as immoral. Students in that cohort did not feel they experienced a truly comprehensive curriculum. Simultaneously, only 36% of teachers reported training on all topics of a comprehensive curriculum [7].

A related study in Kisumu, Kenya revealed that students reported a high level of stigma surrounding contraceptive use and abortion [8]. The stigma surrounding reproductive health is poorly understood, and teachers may play a role in perpetuating stigma that surrounds adolescent sexual health, premarital sex, contraceptive use, and pregnancy and abortion. When a woman seeks an abortion, others may see her as inferior or devalue her worth [9,10]. Understanding the stigma may lead to a better understanding of how to decrease barriers to access for contraception, abortion, and reproductive health information. In turn, improving access could help prevent unplanned pregnancies and decrease morbidity and mortality of unsafe abortion. This study aimed to investigate the stigmatizing attitudes toward girls associated with contraceptive use and/or abortion among secondary school teachers in Kenya.

## 2 Materials and methods

This cross-sectional study investigated the stigmatizing attitudes of teachers toward abortion and contraception. The project was conducted in partnership with Kisumu Medical & Education Trust (KMET), a local NGO, and the Kisumu County Ministry of Education and Ministry of Health. This analysis represents a sub-study with teachers from 4 secondary schools, nested within a larger study assessing stigma surrounding contraceptive use and abortion among secondary school students, teachers, and health care providers (ClinicalTrial.gov: NCT03065842). The inclusion criteria for the schools were as follows: semi-urban secondary day schools, mixed gender, and a minimum of 400 students [8]. The schools all had religious affiliation, which is true for many schools in this region of Kenya.

The Contraceptive Use Stigma (CUS) scale and the Adolescent Stigmatizing Attitudes, Beliefs and Actions (ASABA) scale were used to assess stigma toward girls associated with contraceptive use or/and abortion, respectively [11]. The CUS is a 7-item scale and the ASABA is an 18-item scale, both using Likert scales (1= strongly disagree, 5= strongly agree); both have been validated [11,12]. Higher scores are interpreted as higher levels of stigma. The ASABA has 3 subscales: *Negative stereotypes, exclusion and discrimination*, and *fear of contagion.*

KMET research staff distributed the written scale-questionnaires in January 2018 to all 144 eligible teachers at the 4 suburban secondary schools in Kisumu, Western Kenya. All respondents provided written consent; the consent documents were in English. The Jaramogi Oginga Odinga Teaching and Referral Hospital Ethical Review Committee and the Kenyan National Commission for Science Technology and Innovation provided ethical approval. KMET research assistants administered paper questionnaires, were available for questions, and collected responses in sealed envelopes. All responses were kept confidential and anonymous.

We collapsed the responses as do not agree (1–2), unsure (3), and agree (4–5), and considered responses 3–5 as stigmatizing responses. The scale averages were calculated by dividing the sum of the responses to related questions by the number of questions. We first calculated descriptive statistics and then performed bivariate analyses, comparing scales based on gender and age group using a 2-sample t-test or analysis of variance test, chi-squared test, or Fisher's exact test. To examine the correlation between the CUS and ASABA scales, we used the Pearson correlation coefficient. All statistical analyses were performed on SAS 9.4 (SAS Institute Cary, NC.). Statistical significance was set at *p <* 0.05, using 2-tailed tests. The questionnaires provided an option to leave a comment at the end. We grouped those responses by scale and subscales.

## 3 Results

Of 144 eligible teachers, 122 participated (84.7%). Table 1 shows the characteristics of participating teachers including age, sex, and scoring for contraception and abortion stigma. Overall, the mean age for females was 34.4 vs 38.6 in males (*p =* 0.045).

**Table 1:** General characteristics of secondary school teachers (*n =* 122) in four suburban secondary schools in Kisumu, Western Kenya

	*n*	%*	Mean ± SD	Min	Max
Age	104	-	36.4 ± 10.8	21.0	70.0
≤29	40	38.5	-	-	-
30–39	26	25.0	-	-	-
≥40	38	36.5	-	-	-
Sex	-	-	-	-	-
Female	67	57.3	-	-	-
Male	50	42.7	-	-	-
Contraceptive use stigma⁎⁎ (7 questions, max possible score 35)	-	-	-	-	-
Total score	120	-	17.8 ± 5.2	7.0	31.0
Score per question	120	-	2.6 ± 0.7	1.0	4.4
Abortion stigma⁎⁎⁎ (18 questions, max possible score 90)	-	-	-	-	-
Total score	109	-	38.4 ± 9.0	20.0	62.0
Subcategory total score					
Negative stereotypes (8 questions, max possible score 40)	111	-	23.2 ± 7.0	10.0	40.0
Exclusion and discrimination (7 questions, max score 35)	120	-	11.1 ± 3.2	7.0	24.0
Fear of contagion (3 questions, max score 15)	119	-	4.0 ± 1.4	3.0	9.0
Score per question	109	-	2.1 ± 0.5	1.1	3.4
Subcategory score per question					
Negative stereotypes	111	-	2.9 ± 0.9	1.3	5.0
Exclusion and discrimination	120	-	1.6 ± 0.5	1.0	3.4
Fear of contagion	119	-	1.3 ± 0.5	1.0	3.0

⁎ 1= strongly disagree, 5= strongly agree, score ≥3 stigmatizing.

⁎⁎ Contraception Use Stigma Scale.

⁎⁎⁎ Adolescent Stigmatizing Attitudes, Beliefs, and Actions (ASABA) scales.

Table 2 shows that 52.3 (43%) of teachers reported stigmatizing attitudes toward a girl using contraceptives. Many teachers (*n =* 69; 56.6%) were of the opinion that married women are more deserving of contraception than unmarried women and 69 (57%) were concerned that contraceptive use will impair future fertility. Contraceptive use was associated with promiscuity (*n =* 52; 43%), which could influence peers to a promiscuous lifestyle (*n =* 62; 51%). Most teachers, however, believed that a girl should be able to decide whether to use a condom or not, with only 9 (7.4%) displaying high stigma scores for this item. Sub-analysis revealed no significant differences in the scores with regard to the teacher's age and sex (data not shown).

**Table 2:** Contraception stigma among secondary school teachers (*n =* 122) in suburban secondary schools in Western Kenya.

Contraception stigma*	Stigmatizing scores⁎⁎*n* (%)	Mean ± SD
1. A married woman is more deserving of a contraceptive method than an unmarried woman	69 (56.6)	3.0 ± 1.4
2. A girl who uses a contraceptive method is promiscuous	52 (43.0)	2.6 ± 1.4
3. A girl who uses a contraceptive method will encourage others to a promiscuous lifestyle	62 (51.0)	2.8 ± 1.3
4. A girl cannot decide for herself if to use a contraceptive method	60 (50.0)	2.8 ± 1.4
5. A girl who uses contraceptives will have problem when she decides to get pregnant	69 (57.0)	2.8 ± 1.3
6. A girl who carries condoms is likely to have many sexual partners	45 (37.0)	2.5 ± 1.3
7. A girl should not insist to use a condom. It is the for the man to decide whether to use a condom or not	9 (7.0)	1.4 ± 0.8

⁎ Contraception Use Stigma scale.

⁎⁎ 1= strongly disagree, 5= strongly agree, score ≥3 stigmatizing.

On average, 27.2% of teachers displayed stigmatizing attitudes toward girls associated with abortion (Table 3). The sub-scale *negative stereotyping* (items 1–8) displayed the highest stigma scores (2.90 on average per question) compared to subscales of exclusion and discrimination (items 9–15; 1.59 on average per question), and fear of contagion (items 16–18; 1.33 on average per question). Most teachers believed that a girl who had an abortion is committing a sin (*n =* 90; 73.8%), that her health will never be as good as it was before the abortion (*n =* 88; 72.7%), that if a girl has an abortion, it will become a habit 62 (51.2%), and that having an abortion may influence others to do the same (*n =* 76; 62.8%). Overall, male and female teachers had similar scores, with the average abortion stigma score per question of 2.1 for both. The youngest teachers reported the highest scores regarding abortion being shameful to a woman's family (<29, 64%; 30–39, 39%; ≥40, 39%; *p =* 0.046). Older individuals were more likely support the statement that a man should not marry a woman who had an abortion because she may not be able to bear children again (≤29 years= 7.5%; 30–39 years= 0%; ≥40 years= 18.4%; *p =* 0.045).

**Table 3:** Abortion stigma among secondary school teachers (*n =* 122) in suburban secondary schools in Western Kenya.

Abortion stigma*	Stigmatizing scores⁎⁎*n* (%)	Mean ± SD
1. A woman who has an abortion is committing a sin	90 (73.8)	3.7 ± 1.3
2. Once a woman has one abortion, she will make it a habit	62 (51.2)	2.9 ± 1.3
3. A woman who has had an abortion cannot be trusted	40 (33.6)	2.3 ± 1.2
4. A woman who has had an abortion brings shame to her family	57 (47.9)	2.9 ± 1.4
5. The health of a woman who has had an abortion is never as good as it was before the abortion	88 (72.7)	3.5 ± 1.4
6. A woman who has had an abortion might encourage other women to get abortions	76 (62.8)	3.2 ± 1.3
7. A woman who has had an abortion will be a bad mother	25 (20.8)	2.0 ±1.2
8. A woman who has had an abortion brings shame to her community	51 (42.1)	2.6 ± 1.3
9. A woman who has had an abortion should be prohibited from going to religious services	2 (1.6)	1.3 ± 0.6
10. I should tease a woman who has had an abortion so that she will be ashamed about her decision	7 (5.7)	1.4 ± 0.9
11. I would disgrace a woman in my community if she has had an abortion	7 (5.8)	1.5 ± 0.8
12. A man should not marry a woman who has had an abortion because she may not be able to bear children again	10 (8.3)	1.6 ± 0.8
13. I should stop being friend with someone if I found out that she has had an abortion	1 (0.8)	1.4 ± 0.5
14. I would point my fingers at a woman who has had an abortion to let others know what she did	4 (3.3)	1.4 ± 0.7
15. A woman who has an abortion should not be treated the same as everyone else	45 (37.2)	2.6 ± 1.6
16. A woman who has had an abortion can make other people fall ill or get sick	3 (2.5)	1.3 ± 0.5
17. A woman who has had an abortion should be isolated from other people in the community for at least 4 weeks after having an abortion	8 (6.7)	1.4 ± 0.7
18. If a man has sex with a girl who has had an abortion, he will become infected with a disease	(12.3)	1.4 ± 0.8

⁎ Adolescent Stigmatizing Attitudes, Beliefs and Actions (ASABA) scale.

⁎⁎ 1= strongly disagree, 5= strongly agree, score ≥3 stigmatizing.

The Pearson correlation coefficient between contraception use stigma (CUS) and abortion stigma (ASABA) suggests that they are moderately correlated (*r =* 0.36; *p* < 0.001).

Forty-one respondents provided 51 comments regarding abortion and/or contraception (Appendix 1). Some teachers encouraged the use of contraception, and some did not as they believed it could be harmful for the girl. Abortion was considered a sin by many, but others were more forgiving and thought the girls should be counseled to not make it a habit. Some stated that there are various reasons that can make a girl chose an abortion, such as unexpected pregnancy, pregnancy due to incest and rape, or medical reasons. It was also stated that in some communities that if a woman has an abortion she is supposed to be separated from the community. Others believed that such fears of contagion may not have basis in the real-life situations. One teacher reported that a girl that attempted to have an abortion was expelled. Another cited culture and tradition as the greatest stumbling block to free thought on abortion.

**Table A1:** Optional comments regarding contraception and abortion made by secondary school teachers (*n =* 122) in suburban secondary schools in Western Kenya

**Contraception**
*Use of contraceptives should be encouraged.*
*There is a high level of stigma surrounding use of contraceptives.*
*The 3 months injection (contraceptive) has problems. Many mothers have difficulties after long use.*
*Young girls should be educated on the use of contraceptives before they make decisions on use.*
**Abortion**
***Negative stereotyping***
*A girl who had an abortion should be loved and accepted not hated and condemned.// Should not be discriminated but should be counseled. // A woman should have the option of aborting or not, without being judge or criminalized.// Maybe she had no option other than abortion. She should not be condemned in any way. She should be counseled instead of being disgraced.*
*They too deserve a better treatment and a second chance. Proper education to be given the girl on the effect of abortion to avoid being a victim and being depressed.*
*A girl who has had an abortion should be treated normally like any other girl except she can be counseled not to make it a habit. // Abortion is a sin but there are various reasons that can make one abort, such as unexpected pregnancy, pregnancy out of incest, rape, etc. One can abort on medical grounds.*
*Abortion is committing a sin to God. // Abortion is murder and murder is sin. Everyone should flee from sexual sin.// Abortion is a sin and should not be thought of by anybody. Moreover, children in God's eye are blessings. So the world should accept children born out of wedlock!!// It's a sin to abort, young girls must be counseled. Abortion is not accepted in our community. Girls should abstain until wedlock!// Jesus came for the sinners. Repentance heals. Repentance heals even the worst of sinners. Acts 17:30. John 3:16. ?*
*Abortion is a crime unless otherwise.*
*In my community abortion is viewed very negatively.*
*Abortion is a woman's right. // Abortion is one's choice.*
*My community does not support abortion but we cannot judge.*
*Abortion may be necessary if, one is raped, incest has taken place, or has ill health.// There are reasons that may justify abortions like rape, incest, age and health conditions.// Abortion should be discouraged but accepted only when there is complications when giving birth.// The consequences of abortion might be unbearable but abortion should be allowed for medical reasons if they can afford the lady/girl a good survival rate.*
*Abortion is to be conducted when it is absolutely necessary or when there is a good cause. As long as it can promote and enhance quality health in the community. Abortion should be done with democracy?? and with one´s consent. Girls, parents, teachers and students should be made or sensitized to make an informed decision on abortion*
*Abortion should be strongly discouraged especial at a tender age of a girl child.// The abortion should be discouraged at all cost.// Abortion is not a good practice. Women should be encouraged to have babies once they have conceived.*
*Girls should always be canceled so that they don't engage in abortion and sexual activities before marriage.*
***Exclusion and discrimination***
*You know what happens in our schools if a girl is found to have attempted abortion in school?? she´s expelled it happened this term!*
*Instead of discriminating against a woman/girl who has had an abortion, she should be taken for counseling you make her understand the problems (medical) associated with abortion.* *In my community, if a woman has an abortion she is supposed to be separated until when the time is right for her to join the rest of the clan members. That's how it's done. She cannot eat with pregnant women or carry infants.*
*The exclusion and discriminations stated above may not transform the girls who has aborted*
***Fear of contagion***
*These fears of contagions may not have basis in the real-life situations.*
**Other**
*More guidance need on this topic.// There is a need for awareness about abortion.*
*The healthy sex life should be deserved by all gender at equal capacity.// Matter sexual should be an agreement between the 2 parties (boy and girl). No party is superior.*
*All the items depend on the social and economic upbringing of an individual.*
*Culture and tradition are the greatest stumbling block to free thought on abortion.*
*The best guidance is the one which comes from the word of God coupled with parental guidance and peer guidance based on scripture.*
*Abstinence should still be encouraged as a desirable value/status amongst our young girls and boys.*

Comments were optional at the conclusion of the survey. There were 41 teachers that commented with 51 total comments, which were grouped by scale and subscale. Comments that were irrelevant (i.e., those pertaining to the survey in general) were not included above and comments that were redundant were removed. Similar topics are separated by //.

## 4 Discussion

Stigma regarding contraceptive use and abortion was common among secondary school teachers. These stigmatizing attitudes may well hinder efforts toward improving the sexual and reproductive health of teenagers and must be addressed by governmental and nongovernmental agencies that focus on education and health to increase access to effective contraception and decrease unsafe abortion.

This study was nested within a larger study that assessed stigma surrounding contraceptive use and abortion among secondary school students, teachers, and health care providers. The part of the larger study that focused on students showed that there were high levels of stigma among the students surrounding contraceptive use and abortion [12]. Comparing the results of the students’ responses from that study with our results shows that the mean contraceptive use stigma score among their students was 19 compared with 18 among our teachers; and the mean abortion stigma score was 46 among students compared with 38 among teachers [12]. Students and teachers have similar contraception and abortion stigma scores and further study regarding the correlation between these 2 groups could be beneficial for designing interventions that reduce stigma. If teachers receive training and education on comprehensive sexuality education that decreases their stigmatizing attitudes, such training may increase the chances to foster a school culture that is less stigmatizing toward those using contraception or undergoing an abortion.

Teachers may be more open to contraception as opposed to abortion, particularly when viewing abortion as a sin. In the data validation, this idea was discussed with the teachers and they were open to learning more about contraception as a means to decrease abortion, as abortion was highly associated with sinful behavior. Given the religious affiliation of all the schools, it is not surprising that abortion is seen as a sin.

Certain findings within the results are encouraging; we found little stigma in the abortion stigma sub-scales related to fear of contagion and exclusions and discrimination. Contraception is an effective measure to prevent unintended pregnancies and thereby unsafe abortion. When the use of contraception is stigmatized, the prevention of unsafe abortion can be challenging. In addition, while efforts are being made by governmental and by nongovernmental agencies like KMET to improve the safety of legal abortion procedures, high levels of stigma may force young women to secretly seek clandestine abortion procedures outside the health care system.

The prevalence of unsafe and complicated abortion in Kenya may have influenced answers to questions related to health and safety of women who undergo abortion, rather than stigma alone. As abortion care and safety are improved, the safety of abortion would be realized. The results may have limited applicability to other settings outside of this region of Kenya. However, that fact that male and female teachers of all ages were recruited from different schools with anonymous survey responses strengthens the applicability to similar settings in Kenya. In addition, teachers are a rarely studied population and the result may set the stage for future studies within these topics.

Stigma surrounding contraceptive use and abortion was prevalent among these secondary school teachers. The relationship between teachers and students, however, must still be better understood and further studies are needed to determine the best way to reduce such stigma. Evidence-based CSE is one key to increasing SRHR health among adolescents and needs to be free from stigmatizing attitudes. Promoting and protecting adolescent SRHR will lead to great public health, economic, and demographic benefits
